# Supplementary material for: Behavioral demands organize a decision process into distinct yet coordinated neural representations in parietal cortex
Source: bioRxiv. 2026 Sep 18:2026.09.11.750920. Preprint. [Version 1] doi: 10.64898/2026.09.11.750920 (PMC13596226; doi:10.64898/2026.09.11.750920)
Supplement: Supplementary file 1 [file NIHPP2026.09.11.750920v1-supplement-1.pdf]

bioRxiv preprint doi: https://doi.org/10.64898/2026.09.11.750920; this version posted September 18, 2026. The copyright holder for this preprint (which was not certified by peer review) is the author/funder, who has granted bioRxiv a license to display the preprint in perpetuity. It is made available under a CC-BY 4.0 International license.

SUPPORTING INFORMATION

Supplementary figures

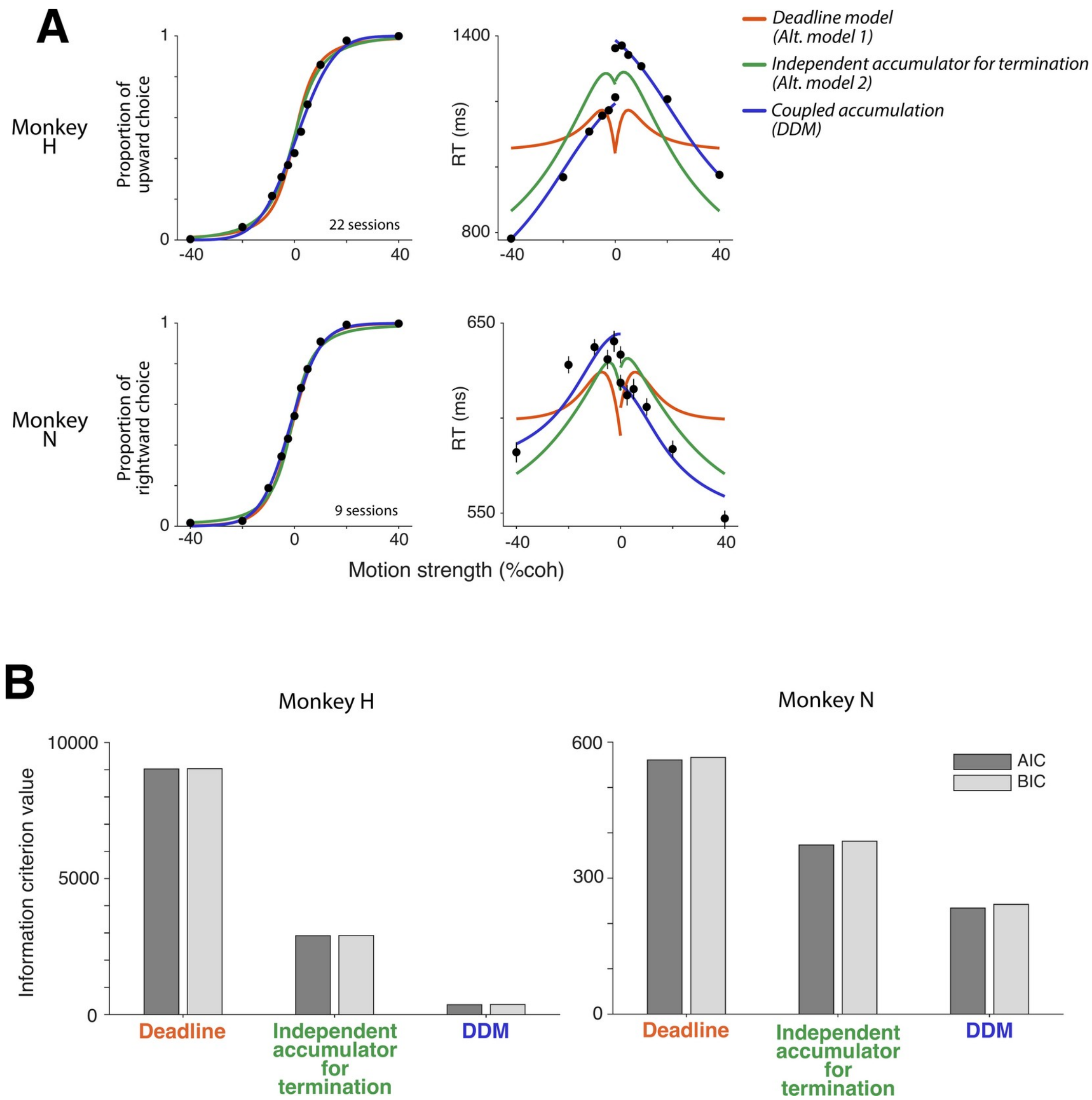

Figure S1. | Comparison of coupled and independent-termination models. A, Psychometric functions (*left*) and choice-conditional chronometric functions (*right*) for monkey H (*top*) and monkey N (*bottom*), together with fits of three models of decision termination and choice: the *Deadline* (Alt. model 1), *Independent accumulator for termination* (Alt. model 2), and *Coupled accumulation* (DDM) models. In the *Deadline* model, evidence accumulates without a bound and termination occurs at an independently generated stopping time. In the *Independent accumulator for termination* model, termination is determined by a separate accumulator driven by unsigned motion strength, while choice is determined by an independent signed-evidence accumulator evaluated at the time of termination. In the *Coupled accumulation* model, signed evidence accumulates to one of two collapsing bounds, with bound crossing jointly determining the time of decision termination and the subsequently reported choice. For the two alternative models, non-decision time was constrained to be the same for the two choices ($t_{nd}^{+}=t_{nd}^{-}$); Figure S2 relaxes this constraint. **B,** Akaike information criterion (AIC) and Bayesian information criterion (BIC) for the three models for monkey H (*left*) and monkey N (*right*). Lower values indicate better model performance after accounting for model complexity.

bioRxiv preprint doi: https://doi.org/10.64898/2026.09.11.750920; this version posted September 18, 2026. The copyright holder for this preprint (which was not certified by peer review) is the author/funder, who has granted bioRxiv a license to display the preprint in perpetuity. It is made available under a CC-BY 4.0 International license.

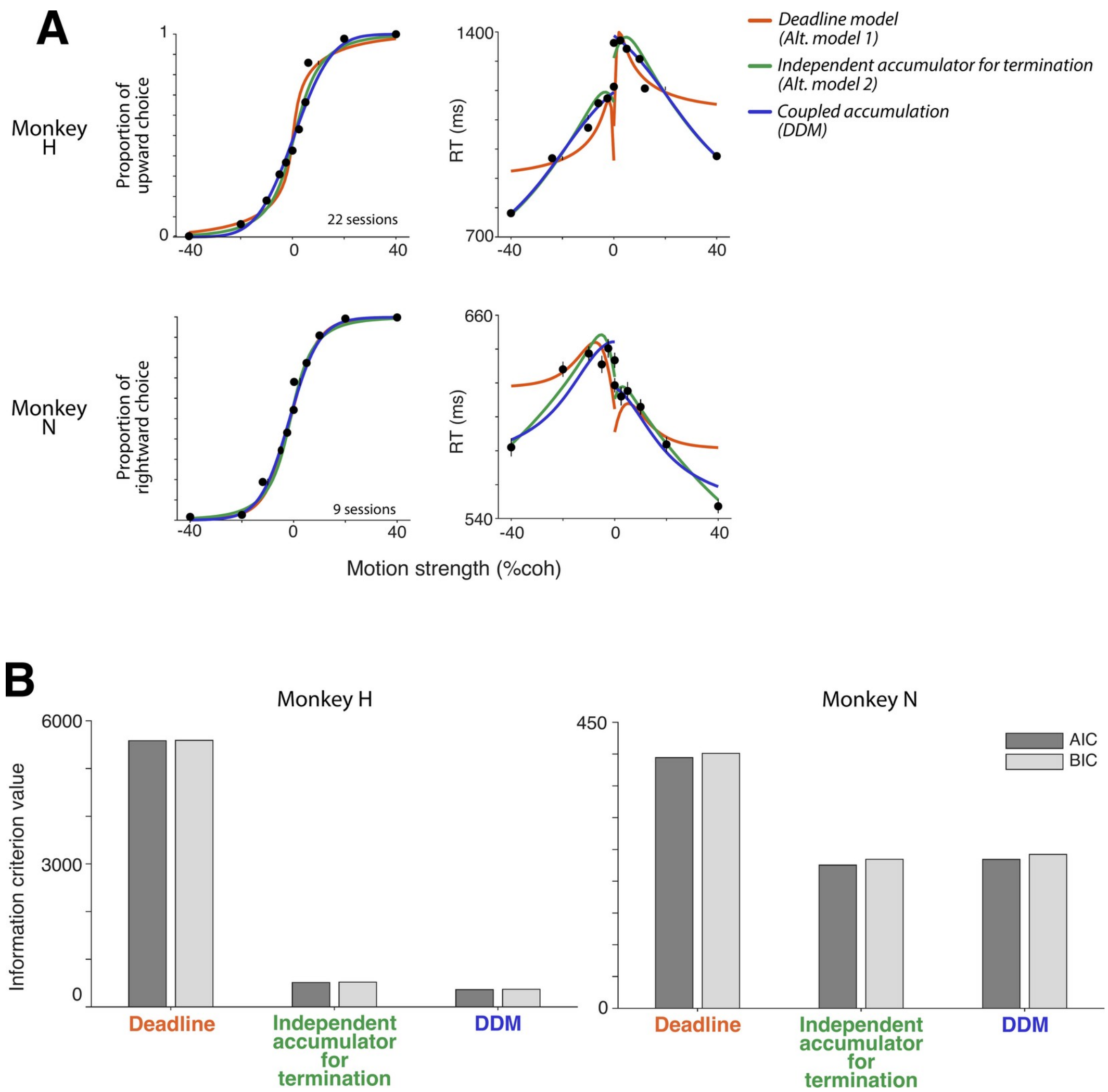

Figure S2. | Model comparison allowing choice-dependent non-decision times in the alternative models. Same model architectures and conventions as in Figure S1 except that the *Deadline* (Alt. model 1) and *Independent accumulator for termination* (Alt. model 2) models were allowed separate non-decision times for the two choices (t_{nd}^{+} and t_{nd}^{-}), matching the flexibility of the *Coupled accumulation* (DDM) model. **A,** Psychometric functions (*left*) and choice-conditional chronometric functions (*right*) for monkey H (*top*) and monkey N (*bottom*), together with fits of the three models. Allowing separate non-decision times provides the alternative models with an additional source of choice-dependent RT differences. **B,** AIC and BIC comparisons for the three models for monkey H (*left*) and monkey N (*right*). With this additional flexibility, the *Independent accumulator for termination* model was modestly preferred for monkey N, whereas the *Coupled accumulation* model remained preferred for monkey H.

bioRxiv preprint doi: https://doi.org/10.64898/2026.09.11.750920; this version posted September 18, 2026. The copyright holder for this preprint (which was not certified by peer review) is the author/funder, who has granted bioRxiv a license to display the preprint in perpetuity. It is made available under a CC-BY 4.0 International license.

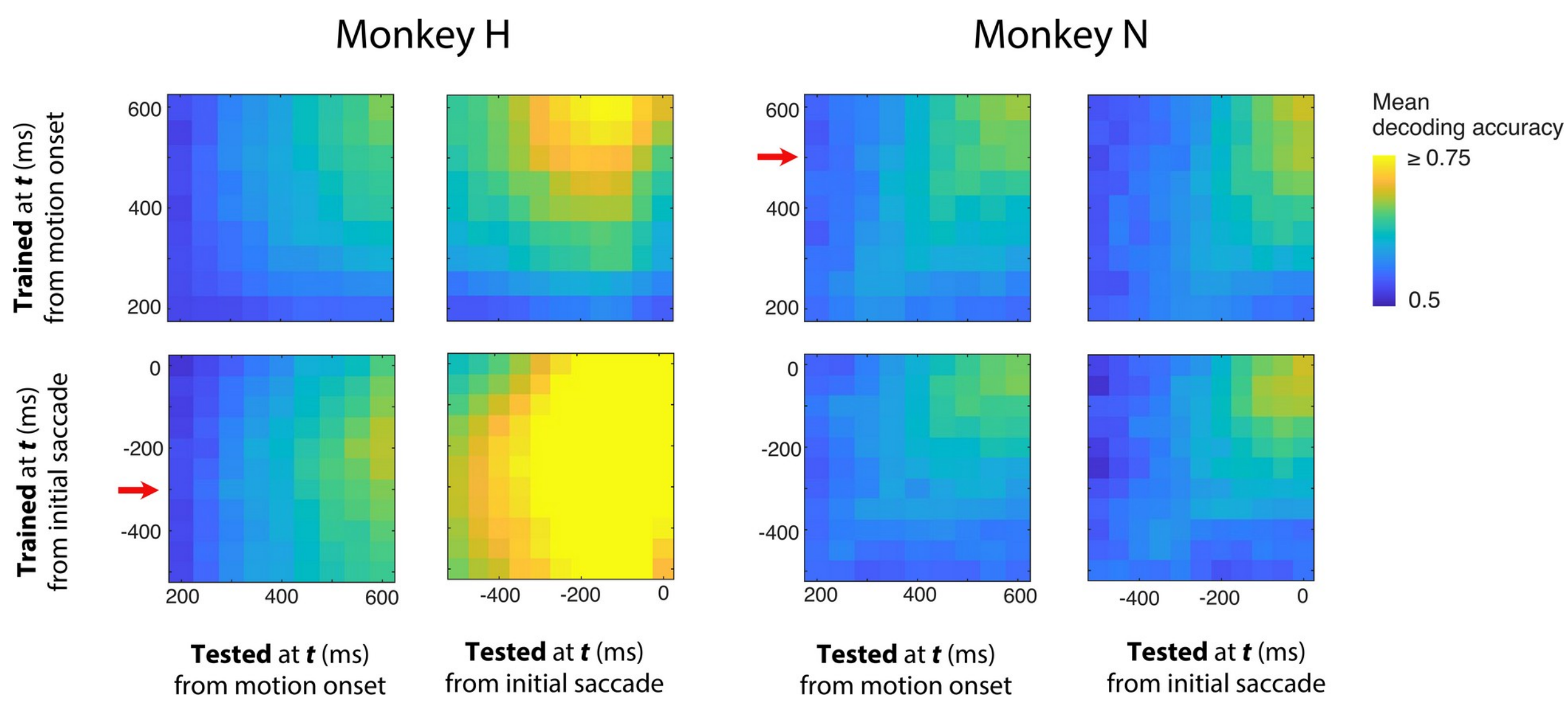

Figure S3. | Temporal generalization of the What coding direction. Temporal generalization of choice decoders trained at different times during decision formation for monkey H (*left two columns*) and monkey N (*right two columns*). Each decoder was trained to discriminate the monkey's eventual choice using population activity within a 100-ms window and evaluated on held-out trials across time. The *top row* shows decoders trained using activity aligned to motion onset and tested on population activity aligned either to motion onset (*left*) or to the T^{when} saccade (*right*). The *bottom row* shows the corresponding analyses for decoders trained using activity aligned to the T^{when} saccade. Thus, each heatmap depicts the generalization of *What* coding directions defined at different training times (vertical axis) to population activity sampled at different test times (horizontal axis). Each pixel shows the decoding accuracy averaged across recording sessions, with sessions weighted equally; chance performance is 0.5. The *What* CD used for subsequent analyses was selected to provide broad temporal generalization across the decision epoch while favoring temporal separation from the T^{when} saccade, thereby reducing potential contributions from peri-saccadic activity. Red arrows indicate the selected training windows for each monkey (monkey H: 300ms before the T^{when} saccade; monkey N: 500ms after motion onset).
